# Ionic Components of Electric Current at Rat Corneal Wounds

**DOI:** 10.1371/journal.pone.0017411

**Published:** 2011-02-25

**Authors:** Ana Carolina Vieira, Brian Reid, Lin Cao, Mark J. Mannis, Ivan R. Schwab, Min Zhao

**Affiliations:** 1 Department of Ophthalmology, University of California Davis, Davis, California, United States of America; 2 Department of Ophthalmology, Federal University of Sao Paulo, Sao Paulo, Brazil; 3 Department of Dermatology, University of California Davis, Davis, California, United States of America; University of Reading, United Kingdom

## Abstract

**Background:**

Endogenous electric fields and currents occur naturally at wounds and are a strong signal guiding cell migration into the wound to promote healing. Many cells involved in wound healing respond to small physiological electric fields *in vitro*. It has long been assumed that wound electric fields are produced by passive ion leakage from damaged tissue. Could these fields be actively maintained and regulated as an active wound response? What are the molecular, ionic and cellular mechanisms underlying the wound electric currents?

**Methodology/Principal Findings:**

Using rat cornea wounds as a model, we measured the dynamic timecourses of individual ion fluxes with ion-selective probes. We also examined chloride channel expression before and after wounding. After wounding, Ca^2+^ efflux increased steadily whereas K^+^ showed an initial large efflux which rapidly decreased. Surprisingly, Na^+^ flux at wounds was inward. A most significant observation was a persistent large influx of Cl^−^, which had a time course similar to the net wound electric currents we have measured previously. Fixation of the tissues abolished ion fluxes. Pharmacological agents which stimulate ion transport significantly increased flux of Cl^−^, Na^+^ and K^+^. Injury to the cornea caused significant changes in distribution and expression of Cl^−^ channel CLC2.

**Conclusions/Significance:**

These data suggest that the outward electric currents occurring naturally at corneal wounds are carried mainly by a large influx of chloride ions, and in part by effluxes of calcium and potassium ions. Ca^2+^ and Cl^−^ fluxes appear to be mainly actively regulated, while K^+^ flux appears to be largely due to leakage. The dynamic changes of electric currents and specific ion fluxes after wounding suggest that electrical signaling is an active response to injury and offers potential novel approaches to modulate wound healing, for example eye-drops targeting ion transport to aid in the challenging management of non-healing corneal ulcers.

## Introduction

At human skin wounds, naturally occurring electric fields and currents were detected many years ago [1]–[4]. Modern techniques have confirmed and significantly extended our understanding of these endogenous electric fields. Electric currents of ∼10–100 µA/cm^2^ and fields of ∼40–177 mV/mm at wounds in humans and various animals have been measured in recent years with vibrating probes, micro-glass electrodes, micro-needle arrays, and bioelectric imagers [5]–[9].

Endogenously induced electric fields at wounds have emerged as an important signal that plays a critical role in wound healing [10]–[13]. Chemotaxis, haptotaxis (response to surface-bound molecules), contact guidance, free edges and other factors at wounds are directional cues for cell migration and wound healing. Recently, we demonstrated that electric signals in cell migration during wound healing are far more important than previously thought since they override other directional cues [6], [10], [14]. Applied electric fields have been shown to stimulate division, migration and differentiation of many types of cells: corneal epithelial cells [15]–[18], neuronal cells [19]–[21], fibroblasts [22] and skin epithelial cells [23]–[24] in a process called galvanotaxis or electrotaxis.

**Figure 1:**
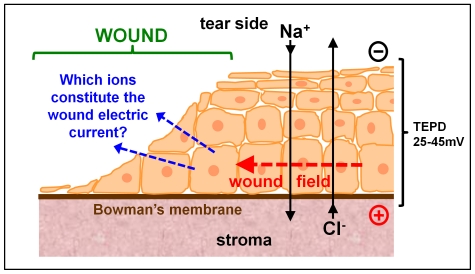
Which ions contribute to the wound electric current? The corneal epithelium transports ions to generate and maintain a transepithelial potential difference (TEPD) of ∼25–45 mV. Injury breaks the epithelial barrier and collapses the potential at the wound (left). The positive potential in the surrounding intact epithlium drives ion current flow out of the wound (blue arrows) and forms laterally-orientated wound electric fields (red arrow) with the wound the cathode.

The mechanisms of how the electric currents are generated and maintained however remain very poorly understood and largely unstudied. It is generally assumed that the wound electric currents are caused by a short-circuit of the trans-epithelial potential difference (TEPD) at the wound site. In another words, a passive leakage of ions and other charged molecules. The TEPD is a common feature of most epithelia, including skin and corneal epithelia. In the cornea, ions are constantly being transported across the corneal epithelium, which generates and maintains the TEPD with the outer side negative [25]. Sodium ions are actively transported from the tears to the aqueous humor while chloride ions are pumped in the opposite direction, towards the tear side (**Fig. 1**) [26]–[28]. Tight junctions between epithelial cells form a high resistance barrier to minimize ion leakage and maintain the TEPD. Damage to the corneal epithelium disrupts the tight junctional barrier and the TEPD collapses at the wound. The electrically positive TEPD under the surrounding intact epithelium thus drives a laterally-oriented electric field running from the intact cornea across the wound edge into the wound center, with the wound the negatively-charged cathode (**Fig. 1**). Human corneal epithelial cells migrate to the cathode *in vitro* [15]–[18] and increasing cornea wound current pharmacologically enhances wound healing [6].

Our own and other studies have shown that wound electric currents have a dynamic time course and appear to be actively regulated [6]–[8]. We thus hypothesize that wound electric currents are an actively modulated wound response mechanism, i.e. wound tissues actively generate and regulate the wound electric currents. We set to investigate the ionic and molecular mechanisms of the endogenous wound electric currents using a cornea wound model. The corneal epithelium has multiple functions including mechanical protection from pathogens and refraction of light onto the lens and retina [29]. Appropriate optical refraction is dependent on a smooth corneal surface and the maintenance of appropriate stromal thickness. As well as the endothelium, the corneal epithelium also contributes to the maintenance of corneal transparency by preventing stromal swelling [30]. The integrity of the epithelial surface is therefore essential for normal vision. Most corneal epithelial wounds are repaired promptly by the migration of epithelial cells at the wound margin towards the center of the wound [31]–[33]. The initial response in corneal wound healing is the migration of surrounding epithelial cells into the wound bed, which is likely to be guided by the electrical signal [6], [10].

Here, we used ion-selective probes to define the ionic mechanisms of wound electric currents. We used pharmacological manipulation of ion transport to alter ion flux, and also fixed the tissues to make sure the fluxes happen only in live tissues. We also determined changes in distribution and expression of chloride transport molecules following wounding. By defining the electrophysiological properties of the cornea we will be able to understand the mechanisms of generation of the endogenous wound electric fields and offer new approaches to manage wound healing and corneal injury. Therapeutic modulation of the corneal wound ion flux in order to enhance the endogenous electric field might represent a promising new strategy towards promoting wound healing in patients with chronic non-healing lesions.

## Methods

### Ethics statement

This study was carried out in strict accordance with the recommendations in the Guide for the Care and Use of Laboratory Animals of the National Institutes of Health. All procedures were approved by the University of California, Davis, Institutional Animal Care and Use Committee (protocol # 07-13078).

### Eyes and custom-made chamber

Using *ex vivo* corneas allows for easier pharmacological manipulation and ion substitution of the bathing solution. We used this model as the predominant experimental system for comparision with our extensive previous data on rat and human donor cornea wound electric currents [6], [34]. The rats (Sprague-Dawley, male, weight 200–224 g) were euthanized by CO_2_ inhalation and cervical dislocation. The eyes were enucleated and kept in cold BSS until use.

**Figure 2:**
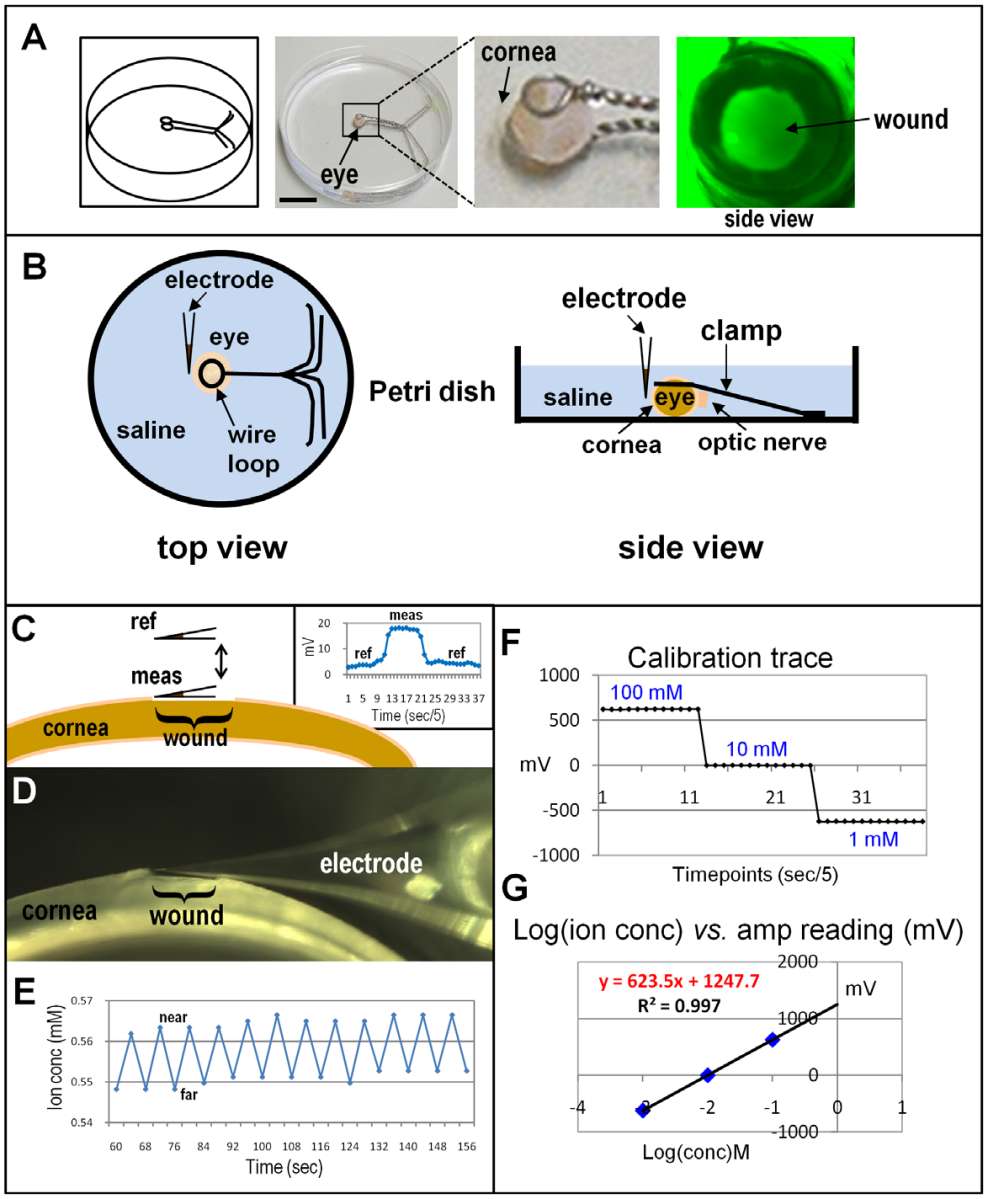
Measuring flux of specific ions at corneal wounds. **A**. Mounting eyes for wounding and probe measurements. A custom-made dish with wire loops holds the eye, leaving the cornea free for measurements and wounding (scale bar 2 cm). Right panel shown wound on cornea stained with fluorescein. **B**. For measurements, the cornea faces the electrode in the center of the dish. The eye can be rotated and/or the electrode moved to measure at different positions. **C**. Schematic showing wounded rat cornea with ion selective microelectrode in reference position (‘ref’) and measuring position (‘meas’) at the wound edge. Insert on right shows raw data recording trace (see Methods for details). **D, E. Self-referencing mode.** **D**. The electrode moves at low frequency (0.3 Hz) between two points (‘near’ and ‘far’) 30 µm apart at the wound edge. **E**. Recording trace which has been converted into ion concentration using the calibration trace (see below). **F, G. Calibration.** **F**. Calibration of the probe in standard solutions. **G**. A linear trendline obtained by plotting the amplifier output in mV *vs.* the logarithm of the ion concentration yields a formula (shown in red) used to calculate ion concentration and, in turn, the actual ion flux.

A chamber was manufactured to hold isolated eyes for wounding and ion flux measurements. This was a 9 cm Petri dish (Falcon; BD Biosciences, San Jose, CA, USA) with two loops of nickel-chromium wire glued in so that the eye is held immobile but can be rotated and/or tilted to give full access to the whole cornea (**Fig. 2A, B**). The diameter of each loop is 5–8 mm. The loops hold the eyeball in place so the cornea can be accessed from different angles. Using a scalpel or a trephine, a wound can be made while the cornea is viewed under a dissecting microscope at ×20–×40. After taking measurements of the unwounded cornea, a wound was made under a dissecting microscope (model SMZ-10, Nikon Instruments Inc., Melville, NY, USA) by scraping the cornea with an ophthalmologic scalpel (Beaver, 22.5°, 4 mm; BD Co., Franklin Lakes, NJ, USA) to remove approximately 2 mm^2^ of corneal epithelium.

### Preparation of ion selective microelectrodes

Borosilicate glass capillaries without filament (10 cm long, 1.5 mm outer diameter, 1.12 mm inner diameter) were purchased from World Precision Instruments (WPI, Inc., Sarasota, FL, USA; cat #TW150-4). Silanization solution I (cat # 85126), sodium ionophore II cocktail A (cat # 71178), chloride ionophore I cocktail A (cat # 24902), calcium ionophore I cocktail A (cat # 21048), potassium ionophore I cocktail A (cat # 60031) and hydrogen ionophore I cocktail A (cat # 95291) were obtained from Sigma-Aldrich (St. Louis, MO, USA). During measurements, corneas were immersed in an artificial tear solution (BSS+; Alcon Laboratories, Inc., Fort Worth, TX, USA) which contained (mM): 122.18 NaCl, 5.1KCl, 1.05 CaCl_2_.2H_2_O, 0.98 MgCl_2_.6H_2_O, 2.96 Na_2_HPO_4_, 25 NaHCO_3_, 5.11 D-Glucose and 0.3 glutathione disulphide.

Borosilicate glass capillaries were heat-pulled using a Sutter P-97 electrode puller (Sutter Instrument Company, Novato, CA, USA) with the following settings: heat 470, pull 13, velocity 15, delay 1, to give tips 3–4 µm in diameter. Electrodes were heated in an oven (Model 10; Quincy Lab Inc., Chicago, IL, USA) at 200°C overnight to dehydrate and then rendered hydrophobic by addition dropwise of about 3 ml of silanization solution. Electrodes were kept in the oven until all the silanization solution had vaporized. After cooling, electrodes were stored in an electrode storage jar (WPI; cat # E215) inside a glass dessicator (Fisher Scientific, Pittsburgh, PA, USA; cat # 08-595D) containing dessicant (Drierite; W.A. Hammond Drierite Co. Ltd, Xenia, OH, USA). To prepare an electrode for use, it was first back-filled using a disposable plastic Pasteur pipette (Phenix Research Products, Candler, NC, USA; cat # PP-137030) heat-pulled to a fine filament, with a 1 cm length of solution containing 100 mM of the ion to be measured. For example, NaCl is used if sodium or chloride are to be measured, CaCl_2_.2H_2_0 for calcium, KCL for potassium, pH 7 buffer for protons. Pressure was applied via a 3 ml syringe with attached silicon tube to push the air-bubble at the tip out, forcing the liquid to the tip. The electrode was then tip-filled with a small amount (50–100 µm length) of ionophore specific for the ion to be measured by attaching the electrode to a micro-positioner and dipping the electrode tip into a droplet of ionophore under a dissecting microscope. Reference electrodes were glass capillaries as above, cut into 5 cm lengths and briefly fire polished in a bunsen burner flame. They were filled with 3 M of NaCl, K-acetate or KCl, with 2% agar. Agar was added to the solution of choice and brought to the boil on a hotplate to dissolve the agar. The hot solution was drawn into the electrode with a plastic Pasteur pipette and the electrode dropped into cold KCl, NaCl or K-acetate solution. Reference electrodes prepared thus can be stored under appropriate solution in Petri dishes for several weeks prior to use. Reference electrodes must not contain the ion to be measured.

Reference electrodes were mounted in a straight microelectrode holder with a gold 2 mm male connector and Ag/AgCl pellet (WPI; cat # MEH3S) and mounted on a 3-dimensional micro-positioner (Newport Corporation, Irvine, CA, USA). Measuring electrodes were mounted in a straight microelectrode holder with a gold 1 mm male connector and Ag/AgCl wire (Warner Instruments, Holliston, MA, USA; cat # QSW-A15P) and attached to a headstage mounted on a computer-controlled electronic 3-dimensional micro-positioner (Newport). The measuring electrode and reference electrode were connected to an Ion Amp V3.0 amplifier (BioCurrents Research Center, Woods Hole, MA, USA).

As well as static measurements of ion concentration (**Fig. 2C**; see section *Selective ion flux measurements* below), electrodes were also used in self-referencing mode, where the electrode moves between two points 30 µm apart at low frequency (0.3 Hz) near the wound edge [35], [36]. The electrode movement was controlled by a computer running IonView32 software (BioCurrents Research Center) controlling the electronic 3-dimensional micro-positioner. The electrode pauses at each position (‘near’ and ‘far’) and the output is recorded to computer (**Fig. 2D, E**). If an ion flux is present, the electrode detects a difference in ion concentration between its two positions. The ion flux can be calculated using the formula: *J = Cu(dc/dx)* where *C* is the ion concentration in the solution, *u* is the ion mobility, and *dc* is the concentration difference over distance *dx*. Ion flux data are presented in *pmol/cm^2^/sec* or *nmol/cm^2^/sec*. Data were recorded onto computer using IonView32 software.

### Calibration of electrodes

Electrodes were calibrated in standard solutions that bracketed the concentration of ion present in the BSS solution (**Fig. 2F**). For example, BSS solution contains ∼150 mM Na^+^, so the sodium electrode was calibrated in 10, 100 and 200 mM NaCl. Plotting the logarithm of the molar concentration against the output of the amplifier (in mV) usually gave a linear fit with an R^2^ value of at least 0.99 (**Fig. 2G**; R^2^ value of 1.0 indicates perfect fit of a linear trendline), and the formula describing the line was used to convert the mV readings from the amplifier during sample measurements into actual ion concentrations from which ion flux can be calculated.

### Selective ion flux measurements

First, the eye was mounted in the chamber (containing BSS saline at room temperature) under the dissecting microscope and allowed to equilibrate to the new temperature. The reference and measuring electrodes were placed in the bath. With the electrode at reference (bath) position approximately 1 cm from the cornea, the background value was recorded for a few seconds. The electrode was then moved into measuring position about 30 µm from the intact, unwounded cornea surface for 10–15 seconds until the new value was stable. The electrode was then moved back to reference position and the recording stopped when the stable background value was reached. The cornea was then wounded as above and measurements made at the left wound edge (**Fig. 2C**). This position was chosen since we previously showed that the current at the wound edge was significantly greater than at the wound center, indicating substantial ion pumping and/or leakage at the cut edge of the corneal epithelium [6]. This gives a single ‘peak’ (see **Fig. 2C** insert on right) and the difference between the background and the wound value in mV can be converted into the difference in ion concentration using the equation derived by plotting the calibration readings (**Fig. 2G**). Wound edge measurements were made at various time-points after wounding to give timelapse data of wound ion concentration change over time. We also did some long time-lapse experiments up to 6 hours.

In self-referencing mode, the oscillating electrode was first placed near the intact cornea to measure unwounded ion flux, and after wounding placed near the wound edge and measurements made in that position for up to 90 min (**Fig. 2D, E**).

### Drug treatments

Aminophylline (cat # A1755) and ascorbic acid (cat # A5960) were obtained from Sigma-Aldrich (St. Louis, MO, USA). We previously showed that drugs which affect ion flux also alter cornea wound electric current and rate of wound healing [6], [34]. Here we tested the effect of aminophylline (10 mM) and ascorbic acid (1 mM) on calcium, sodium and chloride ion flux. Eyes were kept in BSS solution at room temperature with drug for 20 minutes before beginning experiments. We found that the presence of aminophylline interfered with chloride ionophore I cocktail A, perturbing the electrode responses and giving unpredictable results. Therefore, for chloride flux measurements in the presence of aminophylline, we used a different ionophore: chloride-selective liquid ion-exchanger microelectrode cocktail A (Sigma-Aldrich; cat # 24899).

### Data

**Figure 3:**
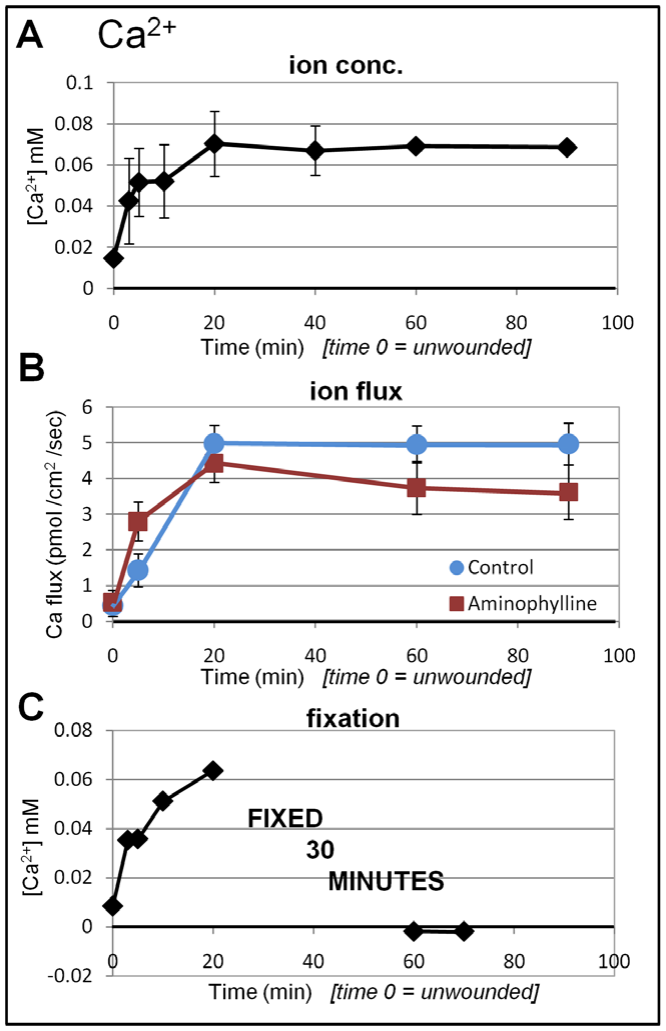
Calcium flux at corneal wounds. **A. Ca^2+^ concentration.** Calcium concentration at unwounded cornea was slightly above background (0.015 mM). After wounding, Ca^2+^ concentraion increased until 20 min then plateaued. **B. Ca^2+^ flux.** Unwounded cornea showed a small Ca^2+^ efflux. After wounding, calcium efflux at the wound edge increased to reach a maximum value after 20 minutes. This efflux was maintained for up to 90 minutes. Aminophylline had no effect on Ca^2+^ flux. **C. Fixation.** Calcium concentration was measured for 30 minutes to confirm normal efflux. The eye was then fixed. Subsequent measurements showed a drop in calcium concentration to almost zero, even lower than unwounded values.

Ion-selective micro-electrode data are expressed two ways. First we show the concentration difference between the reference position in the bath solution, and the measuring position close to the cornea surface or wound edge (see **Fig. 2C**). For example, **Figure 3A** shows calcium concentration at unwounded cornea (time 0) and at wound edge (wound edge has a relatively higher concentration). We then present data of actual ion flux, calculated from data recorded with the electrode in self-referencing mode as it oscillates close to the cornea surface (see **Fig. 2D, E**). For example, **Figure 3B** shows calcium flux before and after wounding, showing a steady increase of calcium efflux (outward ion flow) after wounding. For each ion measured, the concentration difference data, and ion flux data, correlated. For example, higher ion concentration at wound edge = ion efflux (outward flow). Note that in all timelapse graphs (**Figs. 3** **–** [Fig pone-0017411-g004] [Fig pone-0017411-g005] [Fig pone-0017411-g006] **7**), the measurements made at intact cornea surface before wounding are shown at time zero.

### Immunofluorescence

Rat cornea tissue were fixed with 4% paraformaldehyde (Sigma-Aldrich) in phosphate-buffered saline, pH 7.4 (PBS; Sigma-Aldrich) for 20 min. Cryo-sections were cut and collected on gelatin-coated slides. Non-specific binding sites were blocked with PBS containing 2% bovine serum albumin (BSA; Sigma-Aldrich), 10% donkey serum plus 0.3% Triton X-100 (Sigma-Aldrich), for 30 min at room temperature. A monoclonal antibody against CLC2 (1∶200, Santa Cruz Biotechnology, Inc., Santa Cruz, CA, USA; Cat # sc-16430), was used to label the protein (1 hour at room temperature). After washing in PBS, the samples were incubated with Cy3 conjugated secondary antibody (1∶200, Jackson Immuno Research Laboratories Inc., West Grove, PA, USA) and phalloidin-FITC (1∶100, Sigma-Aldrich) for 1 h at room temperature. Cell nuclei were counterstained with 4′,6-diamidino-2-phenylindole (DAPI; Vector Laboratories Inc., Burlingame, CA, USA; cat # H-1200). Flourescent signals were visualized on a fluorescent microscope (Axiovert 100; Zeiss, Germany).

### Quantitative PCR

Expression level of CLC2 mRNA was determined using quantitative real time polymerase chain reaction (qPCR). PCR primers were designed using Primer3 online. Human CLC2 mRNA sequence was downloaded from the National Center for Biotechnology Information (NCBI) GenBank. A region that matched the consensus sequence for CLC2 was chosen for PCR primers. Primers were chosen to be 20 bp in length, with a GC content of 45–60%, no long repeats of a single base, and 150–500 bp of products length. We further confirmed lack of cross-reactivity between the 3′ end of the CLC- primers and other known genes by BLAST (basic local alignment search tool) searches. The optimized primer oligos used were synthesized by Eurofins MWG Operon (London, UK). Primary antibody against CLC2 was obtained from Santa Cruz Biotechnology, Inc. Human corneal epithelial cells were cultured in supplemented hormonal epithelial medium (DMEM/F12; Invitrogen, Carlsbad, CA, USA) in 10 cm tissue culture dishes at 37°C and 5% CO_2_ until confluent. Scratch wounds were made with a sterile plastic pipette tip.

### Vibrating probe

We have previously described in detail the vibrating probe technique for non-invasively measuring endogenous electric current [7], [37]. Briefly, the probe is an insulated stainless-steel needle (WPI; cat # SSM33A70) with a platinum ball electroplated to the tip. The probe, mounted on a 3-dimensional micromanipulator (model H; Line Tool Co., Allentown PA, USA), is vibrated in solution about 50 µm from the cornea surface by a piezoelectric bender (made in-house) at a precise frequency (usually ∼150–200 Hz). If an electric current is present due to ion flux, the charge on the platinum ball fluctuates in proportion to the size of the current. The probe is connected to a lock-in amplifier (model SR530; Stanford Research Systems, Sunnyvale, CA, USA) that locks on to the probe's specific frequency signal. The probe is calibrated in a current density of 1.5 µA/cm^2^ at the start and end of experiments. Eyes were treated as above for 20 min in drug at room temperature before measurements. DIDS (4,4′-diisothiocyanatostilbene-2,2′-disulfonic acid disodium salt hydrate) was obtained from Sigma-Aldrich (cat # D3514).

### Statistics

Data analysis, graphs, linear trendlines and statistical calculations were done with Microsoft Excel. Data are expressed as mean ± standard error of the mean (SEM). Differences between mean values were compared using Student's *t* test.

## Results

We present the data from ion-selective measurement in two ways: 1) as the ion concentration compared to background: higher or lower at the cornea surface or wound edge; and 2) as ion flux, calculated from data recorded with the probe in self-referencing mode oscillating close to the wound edge.

### Gradual and persistent increase in calcium efflux at corneal wounds

We first measured calcium concentration at corneal wounds. Calcium concentration at unwounded cornea surface was only slightly higher than background level in the artificial tear solution (0.015±0.002 mM). After wounding, the calcium concentration increased within the first 20 min. The level then remained stable at around 0.07 mM (**Fig. 3A** **,** showing only up to 90 minutes). In longer experiments, this value was maintained, remaining significantly higher than the unwounded value for up to six hours after wounding (P<0.04).

We then determined calcium flux at corneal wounds using the electrode in self-referencing mode to calculate the actual calcium ion flux. We saw a small calcium efflux in unwounded corneas. After wounding, the calcium efflux increased steadily until 20 min, stabilizing at a maximum value of 5 pmol/cm^2^/sec, that was significantly greater than unwounded flux (P<0.02) (**Fig. 3B**).

We previously showed that pharmacological drug aminophylline significantly increased cornea wound current and enhanced wound healing [6]. Here, aminophylline (10 mM) had no effect on calcium flux (**Fig. 3B**), suggesting that aminophylline-induced enhancement of cornea electric current is not due to increased calcium flux. Amiloride, which inhibits the Na^+^/Ca^+^ exchanger and voltage-gated Ca^+2^ channels [38], also had no effect on calcium flux (data not shown). To confirm that calcium efflux was due to active transport, we measured calcium concentration for 30 minutes to confirm normal efflux and then fixed the cornea for 30 min in 4% paraformaldehyde. After fixation and washing, the calcium concentration had dropped to virtually zero (**Fig. 3C**).

### Sharp rise and rapid decrease in potassium efflux

**Figure 4 pone-0017411-g004:**
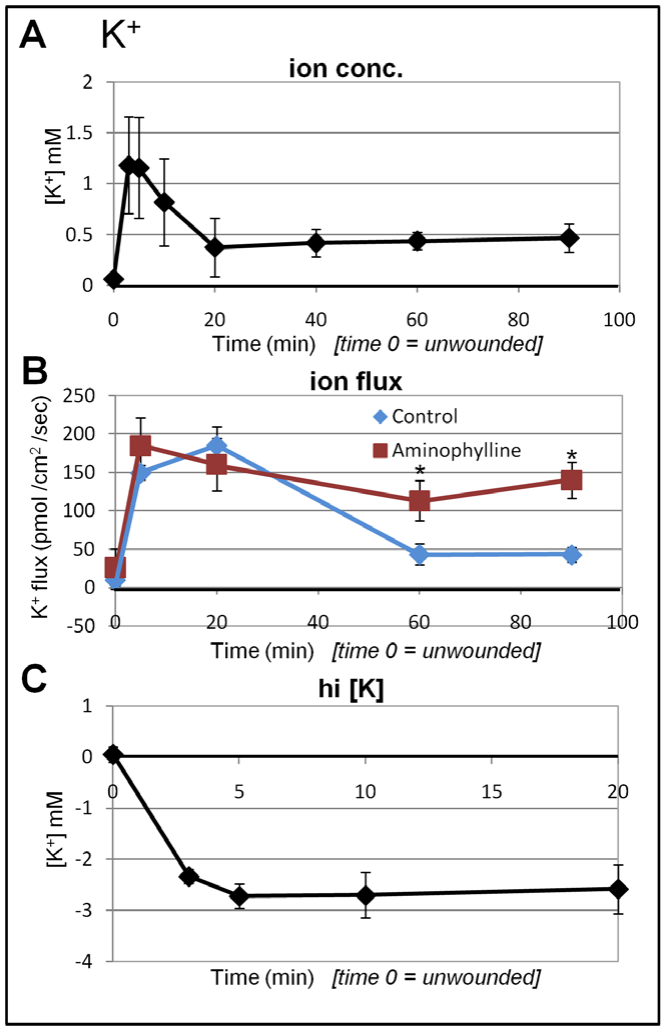
Potassium flux at corneal wounds. **A. K^+^ concentration.** Immediately after wounding there was a rapid transient increase of K^+^ concentration. This initial high concentration decreased over 5–20 min after wounding, reaching a stable lower value. **B. K^+^ flux.** There was a large K^+^ efflux immediately after wounding, which dropped after 20 min. Aminophylline had no effect on the initial peak of efflux, but appeared to enhance efflux at later time points (60–90 min; *P<0.03). **C. K^+^ concentration in high [K^+^]**. In hi-K^+^ solution (20 mM) the initial peak of K^+^ concentration was absent and the wound showed a lower K^+^ concentration (negative values), indicating K^+^ influx.

Potassium concentration at unwounded cornea surface was virtually the same as the background level in the artificial tear solution. However, immediately after wounding there was a rapid and large increase of potassium concentration at the wound edge from ∼0 mM to over 1 mM (**Fig. 4A**). This high level of potassium then dropped quickly, reaching a lower, stable value at ∼0.5 mM from 20–90 min after wounding which remained significantly higher than unwounded values. Similarly, wounding also induced a rapid increase in K^+^ efflux of 150–200 pmol/cm^2^/sec. This large efflux of K^+^ however was short-lived, starting to decrease 20 min after wounding, and reached a significantly lower level of ∼50 pmol/cm^2^/sec (**Fig. 4B**).

To determine if the initial large potassium efflux was due to ion leakage or active transport, we measured K^+^ efflux after two type of treatment: 1) aminophyline drug, and 2) artificial tear solution with a high potassium concentration (20 mM instead of the normal 5 mM). Aminophylline (10 mM) had no effect on the initial peak, but significantly increased potassium efflux at later time-points (60, 90 min; P<0.03), suggesting that the initial large efflux was leakage, while the later smaller potassium efflux (after 20 min) was due to active transport (**Fig. 4B**). In high K^+^ artificial tear solution (20 mM), unwounded cornea surface had virtually the same K^+^ concentration as that in the background bathing solution. However, immediately after wounding, the initial increase in potassium concentration (indicating efflux) seen in normal solution was absent. Instead, we measured a large *decrease* in K^+^ concentration, reaching a peak (nearly 3 mM lower than the background concentration) at the wound with the same time scale (∼5 min) as the increase of K^+^ in normal artificial tear solution. This decrease in K^+^ concentration indicated an *influx* of potassium (**Fig. 4C**; negative values). The influx was maintained up to 20 min (the longest time we measured). These results suggest that the initial large potassium efflux was leakage from damaged cells/tissue, which can be proportionally reversed by increasing the external potassium concentration. Also, we would note that leakage of other ions (Cl^−^, Ca^2+^, Na^+^) from damaged cells was presumably negligable due to their low intracellular concentrations (more than ten times less than K^+^), and so was not detected by our measurements.

### Influx of sodium at wounds

**Figure 5 pone-0017411-g005:**
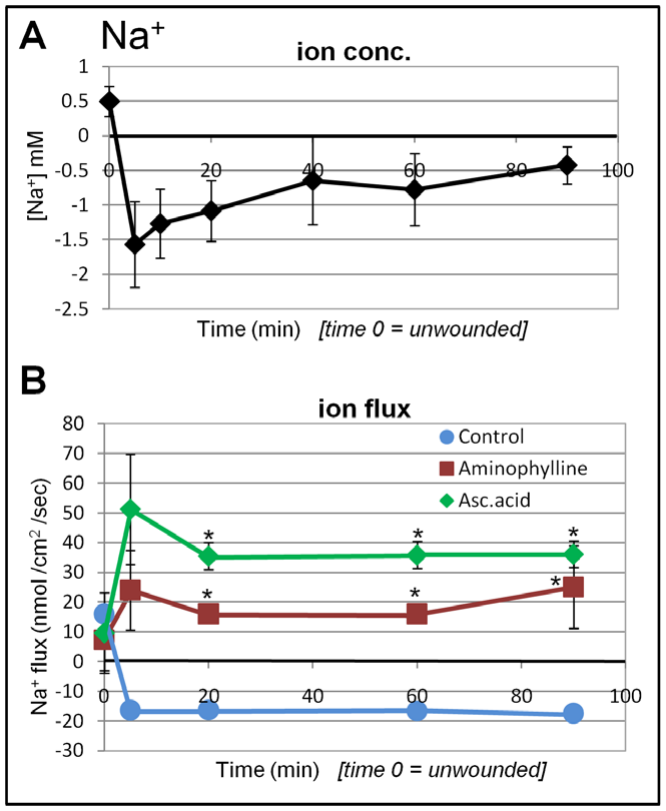
Sodium flux at corneal wounds. **A. Na^+^ concentration.** Unwounded cornea had a slighly higher Na^+^ concentration than background. However, upon wounding, the Na^+^ concentration at the wound edge was lower than background (negative values). **B. Na^+^ flux.** Intact cornea showed a small Na^+^ efflux. On wounding this became a sodium influx which was maintained for 90 min. Both aminophylline and ascorbic acid induced a large Na^+^ efflux at later timepoints (5 min and later; *P<0.01).

Unwounded corneas had a slightly higher sodium concentration than background concentration. Surprisingly, after wounding, sodium concentration decreased and became lower than background (negative values) at the wound edge, reaching the lowest level (1.5 mM) within 5 min (**Fig. 5A**). The sodium concentration difference between the wound and the background started to gradually decrease, but remained significantly lower than background for at least 90 min after wounding.

We then determined the flux of Na^+^ (**Fig. 5B**). Consistent with the changes in Na^+^ concentration at wounds, wounding induced significant sodium influx (15 nmol/cm^2^/sec), which quickly reached a peak in 5 min, and was maintained at the same level for up to 90 min (**Fig. 5B**; negative values show influx).

Because the Na^+^ influx is a very unexpected finding, we tested the effects of two drugs that we have shown previously to increase wound electric currents [6]. Treatment with either aminophylline or ascorbic acid induced significant Na^+^ efflux at the wound. Aminophyline treatment resulted in a significantly increased Na^+^ efflux of 15–20 nmol/cm^2^/sec (P<0.01, when compared to non-drug treated Na^+^ flux). Treatment with ascorbic acid resulted in even higher Na^+^ efflux of 30–50 nmol/cm^2^/sec (P<0.01). The Na^+^ efflux persisted up to 90 min (**Fig. 5B**). In separate experiments, we confirmed these results with concentration measurements: wounds in ascorbic acid had an *increased* sodium concentration rather than the decreased concentration seen in normal solution, confirming the reversal of sodium flux (data not shown).

### Large and steady chloride influx

**Figure 6 pone-0017411-g006:**
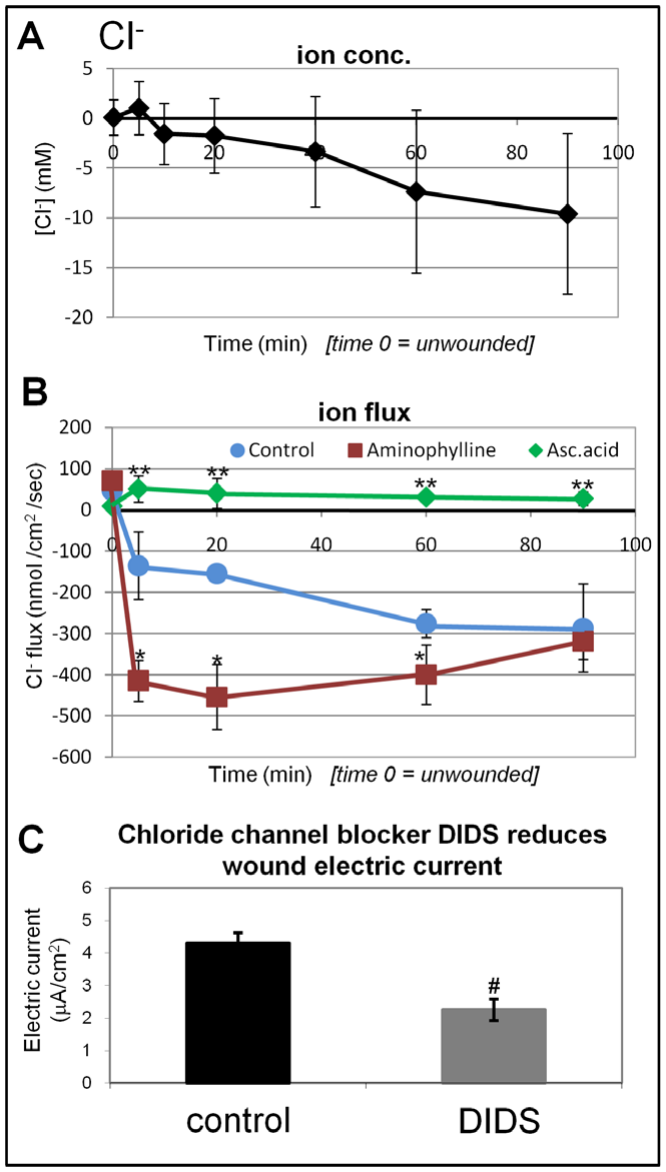
Chloride flux at corneal wounds A. **Cl^−^ concentration.** Unwounded cornea had a slighly higher Cl^−^ concentration than background. Upon wounding, the Cl^−^ concentration at the wound edge transiently increased, (5 min) and then decreased, becoming lower than background (negative values) from 10 min onwards. **B. Cl^−^ flux.** Unwounded cornea had a small chloride efflux. Wounding induced a large, sustained influx which increased with time. Ascorbic acid reversed the chloride influx, giving a small efflux (**P<0.01), but aminophylline significantly enhanced chloride influx at time-points 5–60 min (*P<0.04). **C. Wound electric current** was significantly reduced in the presence of 200 µM broad-spectrum chloride channel blocker DIDS (#P<0.03).

We then focused on Cl^−^ because our previous data [6] indicated a significant role for Cl^−^ in generating cornea wound electric currents. Intact cornea surface showed the same chloride concentration as background. After wounding there was a very slow but steady decrease in chloride concentration at the wound edge (**Fig. 6A**).

Measurement of the Cl^−^ flux at wounds showed a large influx of Cl^−^, largest amongst all the ions we measured. By convention, flux of anion equals electrically the same amount of cation flux in the opposite direction. The Cl^−^ influx thus represents electric current (flow of positive charge) flowing out of the wound. In normal artificial tear solution, Cl^−^ flux quickly reached over 100 nmol/cm^2^/sec (5 min after wounding). The Cl^−^ influx kept rising, reaching a peak of 300 nmol/cm^2^/sec 60 min after wounding and maintained at this level up to 90 min (**Fig. 6B**). The measurement of chloride influx thus correlated with the decrease of Cl^−^ concentration at the wound.

We then tested the effects of aminophylline and ascorbic acid on Cl^−^ flux, because we showed previously that both drugs significantly increased wound electric currents and wound healing [6]. Aminophyline treatment (10 mM) significantly increased Cl^−^ flux from 100–150 nmol/cm^2^/sec to over 400 nmol/cm^2^/sec at 5, 20 and 60 min after wounding (P<0.03) (**Fig. 6B**). Ascorbic acid however appeared to inhibit the chloride influx, although it significantly increased the Na^+^ efflux (see **Fig. 5B** **,** **6B**). To investigate the role of chloride channels in generating cornea wound electric current via active chloride flux, we measured wound electric current with a vibrating probe [7], [37] in the presence of 200 µM broad-spectrum chloride channel blocker DIDS (4,4′-diisothiocyanatostilbene-2,2′-disulfonic acid disodium salt hydrate). DIDS almost halved the wound current (P<0.03) indicating a large contribution of chloride flux to the overall cornea wound electric current (**Fig. 6C**).

### Hydrogen efflux at wounds

We found a small efflux of protons at unwounded corneas, resulting in a decrease of pH of 0.102 pH units at the surface (data not shown). Immediately after wounding there was a significant increase in proton efflux (P<0.02) causing a reduction of pH at the wound edge of 0.605±0.04 pH units. The pH did not change significantly up to 90 min after wounding (P>0.40), staying at about 0.534 pH units, showing a steady and maintained efflux of protons after wounding.

### Relative contributions of different ion fluxes to the overall wound electric current

**Figure 7:**
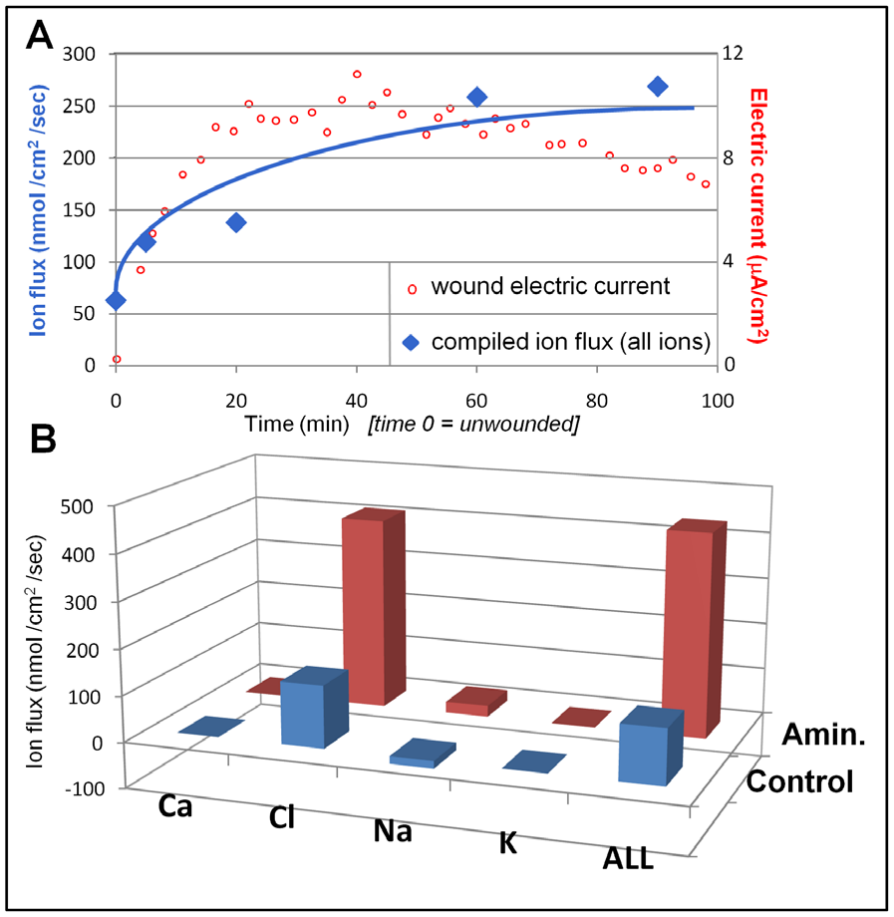
Relative contributions of ions to wound current. **A. Timelapse.** Overlaying wound electric current data (red) with compiled ion flux data (flux of all ions combined; blue) shows that electric current increase after wounding is due to an increasing ion flux (mainly chloride). Wound electric current data from Reid *et al.* [6]. **B. Combined ion flux.** We compiled ion flux data at 5 min after wounding. Chloride is the major ion contributing to the normal (‘Control’; blue) wound current. Enhancement of wound current by aminophylline (‘Amin.’; red) is mostly due to stimulation of chloride flux, but also partly by reversal of sodium flux (inward to outward).

An influx of negative ions generates an outward flow of positive electric current. Therefore, the progressive increase in ion flux (Cl, Ca^2+^) found in our time lapse experiments is consistent with our published data that showed a progressive increase and maintenance of a large outward electric current at cornea wounds [6]. Overlaying the compiled ion flux data (all ions) with the wound electric current shows the correlation between ion flux increase and increase of electric current after wounding (**Fig. 7A**).

Comparing the relative contributions of different ion fluxes to the overall wound electric current showed a predominant contribution of Cl^−^ influx, and negligible contributions of Ca^2+^, K^+^ or Na^+^ (**Fig. 7B**). We chose the 5 min timepoint as this is close to the time that the wound electric current data in Reid *et al.* [6] was measured. Chloride influx was converted from negative to positive, as influx of a negative ion will produce an outward electric current. Sodium influx data was left negative. We can see in **Figure 7B** that chloride ion flux contributes most to the normal (‘control’) wound electric current. Enhancement of wound current with aminophylline appears to be mainly by stimulation of chloride flux, but also to a lesser extent by reversal of sodium flux (inward to outward). We also showed that broad-spectrum chloride channel blocker DIDS (4,4′-diisothiocyanatostilbene-2,2′-disulfonic acid disodium salt hydrate) significantly reduced the overall wound electric current, suggesting that chloride channels, and flux of chloride ions, contribute significantly to the generation of cornea wound electric currents. (**Fig. 6C**). Thus we confirm our hypothesis that active transport of chloride ions is the major contributer to cornea wound electric current.

### Wounding redistributes and upregulates chloride channel CLC2 at corneal wounds

**Figure 8:**
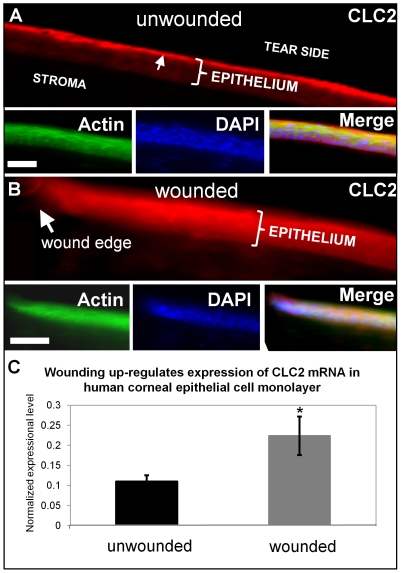
Distribution and expression of calcium-activated chloride channel-2 (CLC2). **A.** In unwounded cornea, CLC2 channels were concentrated in the superficial epithelial cells (arrow). **B.** One hour after wounding, fluorescence was present throughout the entire thickness of the epithelium, showing re-distribution and increased concentration of CLC2 channels. Scale bars 50 µm. **C.** In human corneal epithelial cell monolayer, scratch wounding induced increased expression of CLC2 channel mRNA (*P<0.05).

We then investigated the distribution and expression of Cl^−^ channel CLC2, because it is expressed abundantly and specifically in corneal epithelial tissue [39]. In unwounded corneas, CLC2 channels were seen predominantly in the apical layers of the corneal epithelium (**Fig. 8A**). One hour after wounding (scraping away 2 mm^2^ of epithelium), fluorescence was increased throughout the entire thickness of the epithelium, showing re-distribution and increased concentration of CLC2 channels close to the wound (**Fig. 8B**). Interestingly, the timecourse of up-regulation correlates with the increase of chloride flux, which reached its maximum value one hour after wounding (see **Fig. 6B**). We also used quantitative PCR (qPCR) to detect CLC2 channel mRNA in human corneal epithelial cell monolayer before and after wounding. After scratch wounding, CLC2 mRNA expression was significantly increased (P<0.05) (**Fig. 8C**).

## Discussion

We report here the ionic components of naturally-occurring wound electric currents. Our results challenge the conventional assumption that wound electric currents are merely ion leakage due to breakdown of the epithelial barrier that short-circuits the trans-epithelial potential at the wound site. Using ion selective self-referencing microelectrodes, we demonstrate that wounding the cornea resulted in: 1) a Ca^2+^ efflux that reached a peak of 5 pmol/cm^2^/sec 20 min after wounding and persisted thereafter; 2) a rapid rise in K^+^ efflux to 150–200 pmol/cm^2^/sec that quickly decreased after 20 min following wounding; 3) a surprising influx of Na^+^ of ∼15 nmol/cm^2^/sec; 4) a large and rising Cl^−^ influx reaching 300 nmol/cm^2^/sec that lasted for at least 90 min after wounding. Importantly, the fluxes of different ions had very distinctive time courses, and were abolished or significantly affected by chemical fixation or by pharmacological agents that modulate ion transport. Wounding the cornea resulted in significant re-distribution and up-regulation of CLC2 Cl^−^ channels in the corneal epithelium.

### Trans-epithelial potential difference, wound electric fields and directional signals for cell migration

Epithelia generate electric potentials. The healthy cornea maintains a trans-epithelial electrical potential difference (TEPD) of approximately 25–45 mV, with the tear side negative. Similar TEPs have been found in skin, airway epithelium, gut epithelium, kidney epithelium and other types of epithelia that form tight junctions and transport ions [40]–[46]. Ion transport is required to generate the TEPD. In the cornea, for example, chloride ions are transported from the aqueous to the tear side while sodium is transported in the opposite direction (see **Fig. 1**). Tight junctions between epithelial cells form a barrier of high electrical resistance which helps to maintain the TEPD. Damage or injury to the corneal epithelium breaks this barrier, causing a ‘short-circuit’ at the wound, which produces large and maintained electrical currents [6]. Current flow through adjacent tissue and out of the wound generates endogenous electric fields orientated towards the wound center which stimulate cells to migrate into the wound to promote wound healing [10]. Accumulating experimental evidence suggests strongly that this endogenous electric field guides cells at the wound edge to migrate into the wound center to heal the wound [4], [21], [24], [25], [47]–[49]. Electric fields of physiological strength have very strong guidance effects that override other directional cues in guiding cell migration during wound healing [6], [10].

### Cl^−^ influx constitutes the major part of wound electric currents

The specific ionic fluxes that account for ion transport in corneal epithelia vary among species. For example, in amphibians ion transport in corneal epithelial cells is essentially chloride flux [50], [51]. In mammals, it is accounted for by approximately equal contributions of inwardly directed sodium transport towards the stroma and outwardly directed chloride transport into the tears [30]. We previously postulated that the cornea wound electric current might consist primarily of sodium ions [6], since tears contain high levels of sodium (80–170 mM) [52]. However, in this study we found a small efflux of sodium in unwounded corneas and an *influx* of sodium after wounding. This may suggest that, in rats, sodium ions do not significantly contribute to the net electric current at cornea wounds. The corneas of different species have different mechanisms of ion transport. In rabbits, for example, sodium is transported inward, which is surprising since this does not seem to favor water removal from the stroma [53], [54].

Wounding initiates many signaling cascades. The most well studied include release of growth factors and cytokines and reorganization of extracellular matrix. Cells at the wound actively regulate the release of growth factors and modify the extracellular matrix. With a vibrating probe, we have measured large outward electric currents at cornea wounds, which had a dynamic time course and could be affected by drugs that regulate ion transport, suggesting that the wound electric fields and currents are an active response of cells and tissues to injury [6], [34].

In this study we used ion selective microelectrode measurements to demonstrate that individual ion species, Ca^2+^, K^+^ and Na^+^, also have distinct dynamic time courses at corneal wounds. Calcium efflux started small and progressively increased until reaching a stable value after around 20 minutes. This stable value lasted at least 90 minutes (**Fig. 3A, B**). Chemical fixation eliminated the calcium flux (**Fig. 3C**). These results suggest that calcium flux is likely to be actively-regulated after wounding. In contrast, potassium ions had a large transient efflux immediately after wounding which dropped after the first 20 minutes (**Fig. 4A, B**). The intracellular concentration of potassium is approximately 140 mM, while the concentration of potassium in the bathing artificial tear solution (BSS+) is only 5 mM, and in tears has been variously estimated at 6–42 mM [52]. In high potassium solution (20 mM), the initial peak seen in normal BSS was absent and the flux direction was reversed, suggesting that the initial large potassium efflux is due to leakage from damaged cells at the wound edge.

An unexpected finding is the influx of sodium (**Fig. 5**). It had been previously been assumed that Na^+^ accumulation at the basal side is a major component generating the transepithelial potential difference. Drug treatment with aminiophylline or ascorbic acid reversed the direction of Na^+^ flux, which may contribute partially to the effects of this drug on wound electric current and healing rate that we have seen previously [6]. In the corneal epithelium, pH can affect barrier function and ion transport [55]. Immediately after wounding, we saw an increase in proton efflux and a subsequent decrease in pH at the wound edge. Decreased extracellular pH has been shown to slow epithelial chloride secretion from stroma to tears [56]. This might partly explain the influx of chloride we saw that progressively increased after wounding.

The large influx of chloride at the wound edge represents a most significant finding (**Fig. 6A, B**). There are four novel features: 1) the Cl^−^ influx increased progressively after wounding with a different pattern than Ca^2+^, K^+^ and Na^+^; 2) the flux is the largest of all the ions measured: ∼300 nmol/cm^2^/sec, very much larger than the flux of Na, Ca^2+^ or K^+^; 3) aminophylline, which we have shown to increase wound electric current and wound healing [6], significantly enhanced chloride influx; and 4) broad-spectrum chloride channel blocker DIDS significantly reduced wound electric current (**Fig. 6C**).

### Wounding increased and redistributed Cl^−^ channel expression

Corneal epithelium has multiple chloride channels (CLCs) and transporters: the CLC gene family, CLCas (Ca^2+^-activated Cl^−^ channels), CFTR (cystic fibrosis transmembrane conductance regulator), and Cl^−^ transporters such as Na^+^-K^+^-2Cl^−^ co-transporter [57], [58]. Some CLCs have been characterized in both rat and rabbit corneal epithelial cells, and more recently in human cornea [39]. CLCa2 plays a role in human corneal epithelial stratification and cell adhesion. During epithelial stratification, hCLCa2 expression level increases, suggesting a role in the growth of multi-layered corneal epithelia during both natural development and tissue cultivation [57], [59]. Here we saw that after wounding CLC2 expression was re-distributed and up-regulated at cornea wound edges (**Fig. 8**). Re-distribution and up-regulation of CLC2 expression and an increase in chloride transport imply that modulation of chloride ion transport is an active response to wounding that plays a role in cellular guidance and migration as well as tissue stratification, which occurs later in the wound healing process, after epithelial cells have covered the entire wound bed [29].

### Pharmacological regulation of fluxes of specific ions

Aminophylline significantly enhanced chloride influx, and stimulated a sodium efflux (**Fig. 5** **,** **6**). Aminophylline is a nonspecific phosphodiesterase inhibitor and enhances cAMP levels, increasing Cl^−^ transport. Cyclic AMP has been implicated in the active transport of chloride in frog [60] and rabbit corneal epithelia [30]. The transparency of frog corneas is influenced by aminophylline, suggesting the participation of chloride in corneal deturgescence [60]. Aminophylline also causes a significant increase in outward electric current in wounded cornea, and significantly increases the rate of corneal wound healing [6]. The effects of aminophyline on Cl^−^ influx and Na^+^ efflux seen here correlate well with our previous observations of aminophylline-enhanced cornea wound currents and wound healing [6].

The corneal epithelium concentrates ascorbic acid to a level that is higher than in any other tissues [61]. Ascorbate is transported into the posterior chamber by the ciliary body epithelium, enters the aqueous humor, diffuses through the corneal endothelium into the stroma and is finally concentrated by the epithelial cells [62], [63]. Ascorbic acid absorbs UV radiation and may provide photo protection to the cornea, lens and retina [53], [64]. Here, ascorbic acid inhibited chloride influx, but caused a large sodium efflux (**Fig. 5** **,** **6**). The decrease in chloride influx might be compensated for by an increase in sodium efflux to maintain the TEP. Ascorbic acid stimulates active transport of both sodium and chloride across amphibian cornea, probably by inhibiting phosphodiasterase activity and thus increasing the cyclic AMP content of the corneal epithelium [62], [65]. Ascorbic acid is known to aid in healing and significantly decreases the incidence of corneal ulcerations and perforations in alkali burned eyes [66]. We demonstrated that ascorbic acid enhances the transcorneal potential difference, wound electric currents and wound healing rate in the cornea [6]. In addition to the effect on phosphodiasterase, ascorbic acid may increase Na^+^ efflux through mechanisms yet to be defined.

These results suggest a very important aspect in wound electric signaling, that is active transport of ions rather than passive flux or leakage. Mechanistically, they point to specific ionic mechanisms. Leakages of ions, into or out of tissues, following breakdown of the epithelial barrier and cell membranes, is undoubtedly an important initial response after wounding. This leakage is however very short-lived, rising to peak in a couple of minutes and returned to almost baseline 20 minutes after wounding. The leakage of K^+^ from damages cells is driven by concentration gradients. Intracellular K^+^ concentration is about 30 time higher than that outside the cell, while intracellular Na^+^, Cl^−^ and Ca^2+^ concentrations are much lower than in the extracellular space. The subsequent concentration gradients dictate that the majority of leakage from damaged cells is K^+^. In contrast, we saw flux of other ions (Cl^−^, Ca^2+^) increasing steadily after wounding, suggesting that active transport of these ions is dynamically regulated as part of the wound response. Clinically, these results point to better targeted manipulation of the long term wound electric signaling through enhancement of Ca^2+^ and Cl^−^ transport to enhance wound healing.

In summary, increased active transport of Cl^−^ is a rapid response to injury and forms a significant portion of the wound electric current. Wounding cornea induced fluxes of Ca^2+^, K^+^, Na^+^, Cl^−^, and H^+^ with distinct time courses, which can be modulated by fixation or pharmacological treatment. Increased Cl^−^ flux after wounding correlated with up-regulation and re-distribution of Cl^−^ channels. Electric signaling at wounds appears to be an active signaling mechanism which may be exploited to induce and accelerate wound healing. Defining the ionic and molecular foundations of wound electric field generation offers potential novel approaches to modulate wound healing, e.g. to develop eye-drops targeting ion transport to aid in the challenging management of non-healing corneal ulcers.
